# Electrical Characterization of Epoxy Nanocomposite under High DC Voltage

**DOI:** 10.3390/polym16070963

**Published:** 2024-04-02

**Authors:** Ammar Alsoud, Samer I. Daradkeh, Saleh R. Al-Bashaish, Adel A. Shaheen, Ahmad M. D. (Assa’d) Jaber, Adel M. Abuamr, Marwan S. Mousa, Vladimír Holcman

**Affiliations:** 1 Central European Institute of Technology, Brno University of Technology, Purkynova 656/123, 61200 Brno, Czech Republic; samer.daradkeh@ceitec.vutbr.cz; 2 Department of Physics, Faculty of Electrical Engineering and Communication, Brno University of Technology, Technická 2848/8, 61600 Brno, Czech Republic; holcman@vut.cz; 3 Department of Basic Sciences, Faculty of Arts and Sciences, Al-Ahliyya Amman University, Amman 19328, Jordan; S.albashaish@ammanu.edu.jo; 4 Department of Physics, Faculty of Science, The Hashemite University, P.O. Box 330127, Zarqa 13133, Jordan; adel_shaheen@hu.edu.jo; 5 Department of Basic Medical Sciences, Faculty of Medicine, Aqaba Medical Sciences University, Aqaba 77110, Jordan; ahmad.jabr@amsu.edu.jo; 6 Department of Renewable Energy Engineering, Jadara University, Irbid 21110, Jordan; adelabuamro@gmail.com (A.M.A.); m.mousa@jadara.edu.jo (M.S.M.)

**Keywords:** DC breakdown strength, epoxy nanocomposites, electrical properties, high voltage, field electron microscopy

## Abstract

This work studies the direct current breakdown characteristics of unfilled epoxy and epoxy nonconductive nanocomposites (${SiO}_{2},MgO$ and ${Al}_{2}O_{3}$). It also examines the variation of electrical properties in epoxy nanocomposites. The novel aspect of this study is that the samples of Epoxy nanocomposite were exposed to high voltages of up to six kilo volts for three hours using field electron microscopy under high vacuum conditions ($10^{-5}$ mbar). The current emitted from these samples was measured at three different intervals of time. In addition, the influence of high voltage on the permittivity, loss factor (tan(δ)), and conductivity of the epoxy nanocomposite was studied. This evaluation was conducted before and after applying the voltage at room temperature, The frequency range extends from $10^{-2}$–$10^{-7}$ Hz using the Novo Control Alpha-A analyzer. Current–voltage characterization was performed through field electron microscopy. The samples were characterized by scanning electron microscopy–energy dispersive X-ray spectroscopy and Fourier Transform Infrared Spectroscopy. The unfilled epoxy exhibited structural degradation, resulting in the formation of holes when exposed to high voltages of up to six kilo volts, leading to a reduction in electrical properties. Nevertheless, the addition of nanoparticles shows a significant increase in the operational lifetime of the epoxy nanocomposite. The degree of increase in the lifetime of epoxy composite varied depending on several factors such as the type of NPs introduced and their respective sizes. The epoxy/${Al}_{2}O_{3}$ nanocomposite comparing with epoxy/MgO and epoxy/${SiO}_{2}$ nanocomposite showed elevated resistance to direct current breakdown strength and maintaining its dielectric.

## 1. Introduction 

Epoxy resin is a dielectric material known for its exceptional electrical, thermal, and mechanical properties, and has established widespread utilization as a matrix material in high-voltage direct current (HVDC) systems and gas-insulated transmission lines (GILs) [1,2]. However, its integrity becomes compromised when subjected to high-voltage conditions [3]. The phenomenon of dielectric performance deterioration in response to an electric field surpassing a critical threshold is commonly referred to as ‘dielectric breakdown’. This critical electric field threshold, leading to material failure, is identified as ‘dielectric strength’ [4]. Dielectric strength refers to the maximum voltage that can be applied to an insulating material before it collapses. Polymeric dielectric materials, under ideal conditions, exhibit an inherent dielectric breakdown strength of up to 8 MV/cm at room temperature [3]. However, in practical applications, this performance degrades significantly, often dropping by several MV/cm due to the presence of defects or impurities [1].

The dielectric strength of epoxy nanocomposites is heavily influenced by the interfacial region, which constitutes a significant volume within these materials [5]. Consequently, the incorporation of nanoparticles (NPs) has the potential to enhance the breakdown performance of epoxy resin. However, the precise mechanism by which breakdown occurs, particularly in relation to interfacial properties, remains unclear [6,7,8,9]. Within the interfacial region of nanocomposites, the dielectric strength is influenced by several influences from various factors, including molecular chain dynamics, carrier traps, and the potential barrier of the polymer nanocomposite structure to charge transfer [6,7].

For numerous applications, the fundamental requirement is the further development of epoxy composites characterized by prolonged service life, facile manufacturability, and cost-effectiveness. Numerous investigations have consistently shown that the incorporation of NPs is an effective strategy for enhancing the thermal, electrical, and mechanical properties of dielectric materials [8]. One of the challenges associated with the utilization of epoxy materials in high-voltage systems is the potential exposure of these materials to electrical discharges [9]. In the scientific literature, the term “corona” is used to describe a perceptible and audible discharge phenomenon resulting from the escalation of localized electric field gradients across a conductor. This escalation results in the ionization and potential breakdown of the surrounding air at that specific point [10,11]. Notably, many of the studies carried out show an augmented resistance to corona discharge in nanocomposite materials as opposed to their micro-composite materials [12,13,14]. Nanofiller particles, when effectively dispersed, result in increased interfacial areas and reduced inter-particle distances. This, in turn, leads to a reduction in the erosion depth of the samples containing these filler particles, thereby enhancing corona resistance [15].

It has been reported in the previous literature that ZnO NPs exhibit superior characteristics at lower filler concentrations, but as the concentration increases, the breakdown strength and other insulation properties decrease due to particle agglomeration [8]. In the case of ${Al}_{2}O_{3}$ NPs, surface treatment mitigates agglomeration, facilitating the even dispersion of particles within the epoxy resin matrix. This, in turn, results in an increase in breakdown strength and other insulation properties, especially at higher filler concentrations [16]. Li et al. [17] observed a significant improvement in the short-term dielectric breakdown strength and partial charge resistance of pure epoxy resin through the inclusion of nano-${Al}_{2}O_{3}$. Furthermore, Wang and Li [18] reported that the epoxy/nano-${TiO}_{2}$ composites exhibit enhanced resistance to alternating current (AC) voltage stress and higher dielectric strength in comparison to epoxy composites containing micron-sized ${TiO}_{2}$.

In contrast to the findings presented in the aforementioned literature, a reduction in the electrical breakdown properties has been observed in epoxy/${SiO}_{2}$ nanocomposites compared to unfilled epoxy [16]. Several studies showed that the main reasons for the significant improvement in the electrical properties are attributed to the distinctive characteristics of NPs and the interface between particles and the epoxy matrix, as demonstrated in prior research [19]. Other studies have investigated the impact of temperature on the electrical performance of nano-MgO/epoxy composites with different mass fractions [20]. These investigations include an examination of both dielectric performance and breakdown performance [20]. On the other hand, Mi et al. [21] incorporated nano-MgO into polyethylene and found that nanocomposites with filler loadings of 0.5 wt.% exhibit a 15.8% higher breakdown strength compared to unfilled polyethylene.

In this study, three types of NPs of different sizes of ${SiO}_{2}$, MgO, and ${Al}_{2}O_{3}$ were used. These composites were subjected to a DC voltage of 6 kV within field electron microscopy (FEM) for 3 h. The emission current was recorded at three different time intervals. Moreover, in this study, the findings were presented by examining the changes in permittivity, loss factor (tan(δ)), and conductivity. All of these contribute to a complete understanding of the electrical behavior of epoxy nanocomposites under high voltage. Furthermore, the study examined the influence of the filler concentration, permittivity, and conductivity on the DC breakdown strength. In addition, scanning electron microscopy–energy dispersive X-ray spectroscopy (SEM-EDS) has been used to characterize the structural transformations occurring in the nanocomposites. SEM micrograph was used to confirm the presence of holes and electrical trees as a result of the breakdown. Fourier Transform Infrared Spectroscopy (FTIR) was used to study the alterations in the carbon bonds inside epoxy.

## 2. Sample Preparation

Ensuring that the filler material is distributed uniformly in the polymer matrix is a foundational challenge in sample preparation. In previous studies, several techniques have been presented for sample preparation [7,20,22,23]. The method that ensures the effective dispersion of filler material in the epoxy matrix was selected. In this study, Epoxylite^®^ E478 (E-478) was used. It was obtained from Elantas (Wessel, Germany). High-purity (99.5% purity) ${SiO}_{2}$, MgO, and ${Al}_{2}O_{3}$ NPs with sizes of 15–30 nm for SiO, 30–50 nm for MgO, and 50–100 nm for ${Al}_{2}O_{3}$ were obtained from Sigma-Aldrich, St. Louis, MO, USA. Different concentrations of the chosen filler were introduced into the E-478 matrix, specifically 3 and 5 wt.%. The epoxy’s viscosity was initially reduced by heating 4 g of epoxy resin at 60 °C for 30 min. Subsequently, NPs were incorporated into the epoxy, mixed manually for 10 min, and dispersed continuously for 4 h with an ultrasonic bath to ensure a distribution that was almost homogeneous. The resulting mixture was then cast into a high-temperature-resistant silicone mold and underwent a two-stage curing process: first, it was cured at 80 °C for 8 h to eliminate the trapped air bubbles, and then it was annealed at 160 °C for 2 h to achieve solidification [22,23,24,25]. The thickness of the prepared samples was approximately 300 μm.

## 3. Characterization: Scanning Electron Microscopy–Energy Dispersive Spectrum (SEM-EDS)

**Figure 1:**
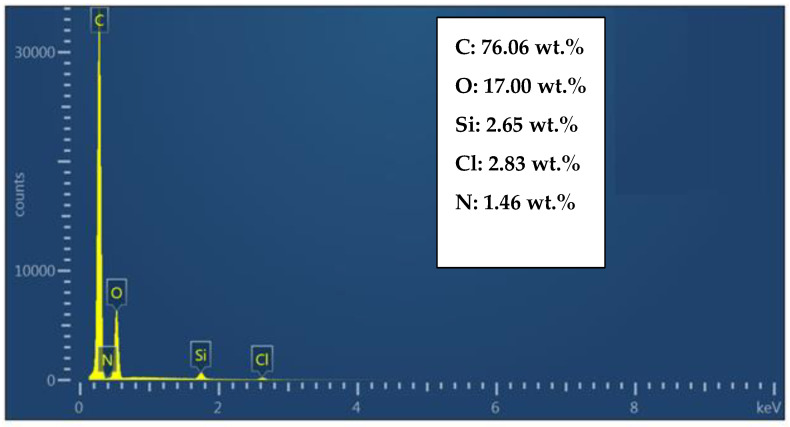
The SEM-EDS analysis spectrum of an unfilled epoxy.

In this study, the composition of the unfilled epoxy resin was analyzed using SEM-EDS equipment (MIRA-TESCAN in the Brno, Czech Republic). Figure 1 shows the SEM-EDS analysis spectra for the unfilled epoxy, revealing the chemical composition of the resin. Primarily, it consists of carbon (76.06%) and oxygen (17.00%), silicon (2.65%) and chlorine (2.83%), and minor amounts of nitrogen (1.46%). It is noteworthy that nitrogen and chlorine are significant components of the amine hardener. The silicone is a thin layer coating that encapsulates the outer surface of the epoxy

**Figure 2:**
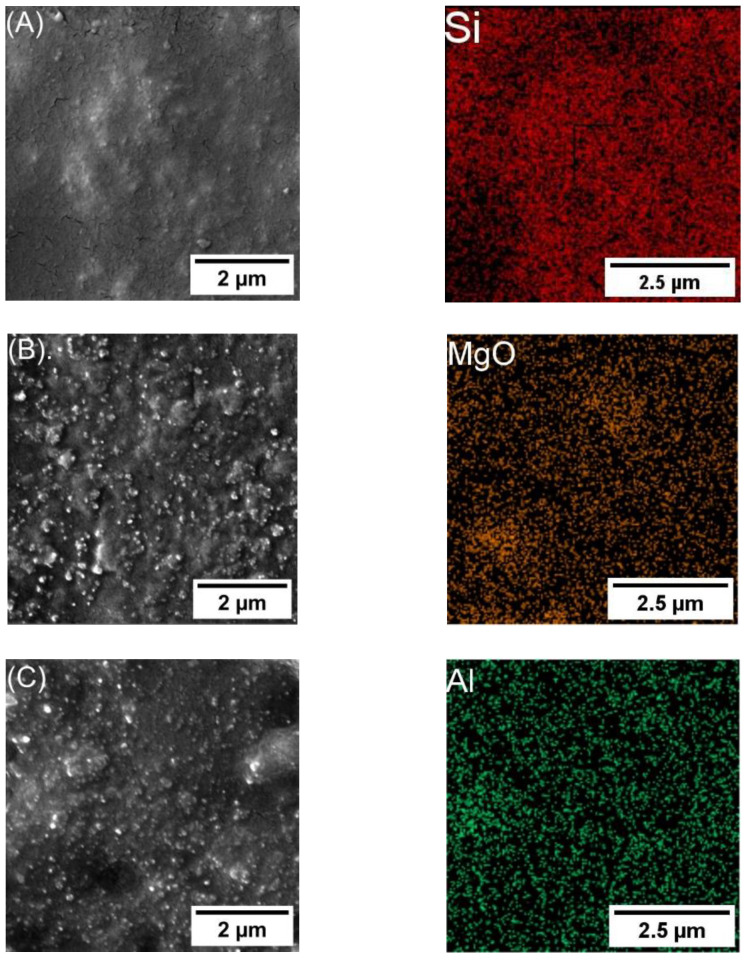
SEM-EDS mapping, including SEM image. (**A**) Epoxy/3 wt.% ${SiO}_{2}$. (**B**) Epoxy/3 wt.% MgO. (**C**) Epoxy/3 wt.% Al_2_O_3_.

**Figure 3:**
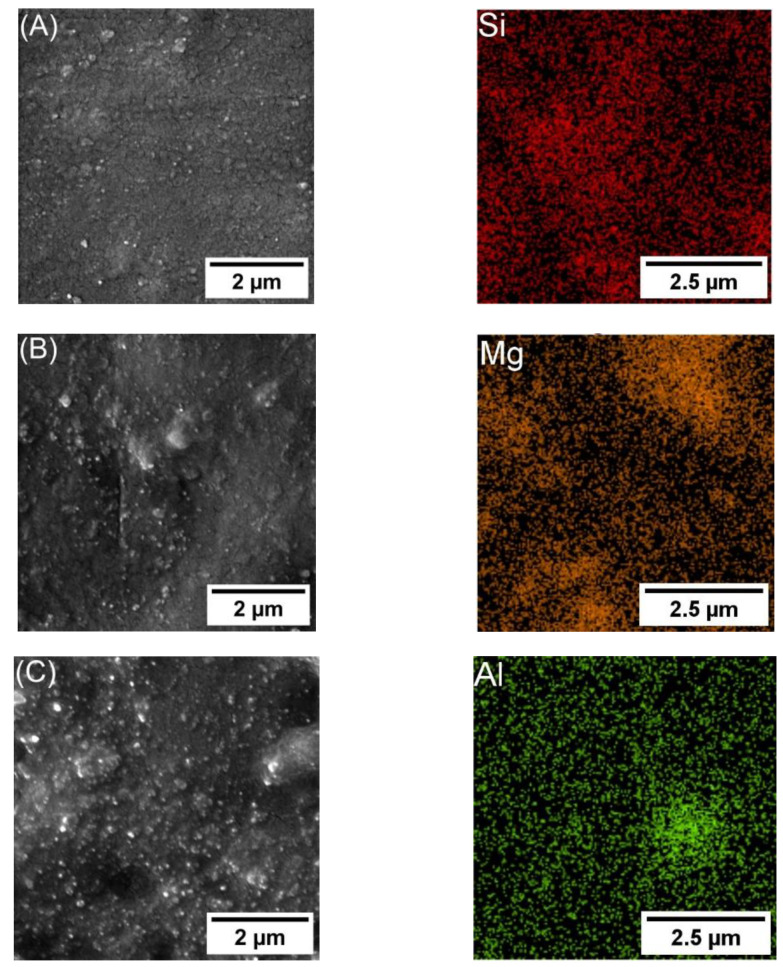
SEM-EDS mapping, including SEM image of Epoxy/5 wt.%: (**A**) Epoxy/5 wt.% of ${SiO}_{2}$. (**B**) Epoxy/5 wt.% of MgO. (**C**) Epoxy/5 wt.% of Al_2_O_3_.

Figure 2 and Figure 3 show the spatial distribution of 3 and 5 wt.%, respectively, of ${SiO}_{2}$, MgO, and ${Al}_{2}O_{3}$ nanocomposite (left) and the NP distribution (right). The spatial distribution was determined through the utilization of the SEM-EDS mapping technique. Moreover, it shows a semi-homogeneous dispersion of NPs in epoxy nanocomposite. Figure 2A shows the SEM-EDS mapping for Epoxy 3 wt.% ${SiO}_{2}$; Figure 2B shows Epoxy/3 wt.% MgO; Figure 2C shows Epoxy/3 wt.% Al_2_O_3_.

## 4. Results and Discussion

This section is divided into two main sections for results from FEM analysis and evaluation of the electrical properties of the epoxy nanocomposite before and after the application of high voltage.

### 4.1. FEM Results

**Figure 4:**
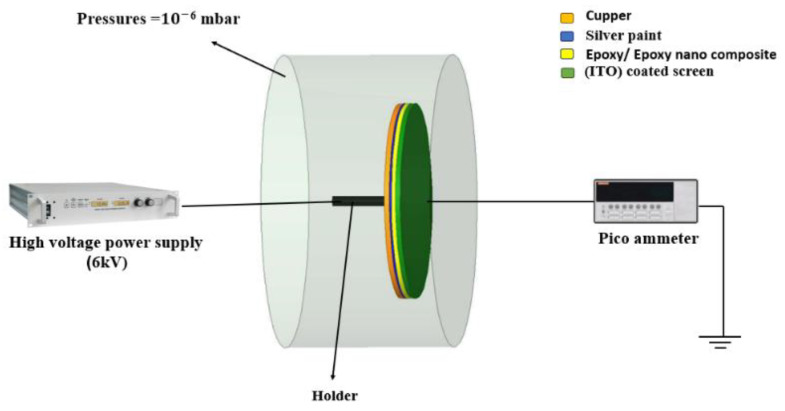
Schematic diagram of the sample within the FEM chamber.

To prepare an appropriate sample for FEM analysis, a broad copper piece with a diameter of 2 cm and a thickness of 1 mm was employed as a substrate for the epoxy nanoparticle. This copper substrate was coated with a thin layer of silver paint to fix the epoxy nanocomposite sample and ensure effective electrical conduction. The copper/epoxy nanocomposite was placed in contact with an Indium Tin Oxide (ITO)-coated screen. In this configuration, the copper/epoxy nanocomposite served as the cathode, while the ITO-coated screen acted as the anode. The FEM system operates at a high vacuum, reaching up to $10^{-6}$ mbar. This high vacuum environment is essential to reduce the rate of ionized gas molecules that backbombard the cathode, thereby protecting it from damage [26]. The samples are connected to a high-voltage power supply with an adjustable range of 0 to 6 kV. The screen is grounded through a precision pico-ammeter, specifically the Keithley 405, to measure the total emission current. During experiments, the voltage was incrementally increased up to 6 kV. The applied electric field had a magnitude of ${2\times 10}^{6}$ V/m. The experiment duration was 3 h. The emission current was measured at three time intervals: at the beginning of the experiment, during the experiment and at the end of the experiment. Figure 4 shows a schematic diagram of the sample inside the FEM chamber. Table 1 shows the emission current values of all samples of the three different time intervals. Table 2 shows the value of the breakdown strength and the time it took for the applied electric field to cause the collapse.

The recorded current measurements, which typically fall within a range of several hundreds pico-amber, provide an overview of the efficiency of the epoxy nanocomposites in trapping electrons. Furthermore, these measurements show the material’s ability to withstand working under the influence of high-voltage conditions. Based on the temporal evolution of current values shown in Table 1, it can be seen that both unfilled epoxy and epoxy/3 and 5 wt.% ${SiO}_{2}$ nanocomposites experienced a significant reduction in emission current over time. Conversely, the epoxy composites enriched with 3 and 5 wt.% MgO NPs have the highest initial emission current values, and they also show a significant reduction by around half of their initial values after 90 and 180 min. Notably, the epoxy composites with NPs of 3 and 5 wt.% of ${Al}_{2}O_{3}$ exhibited a relatively stable emission current during the experiment. However, it showed resistance to collapse despite the applied voltage over time, as shown in Table 2. These findings provide preliminary insights into the potential alterations in the dielectric characteristics of unfilled epoxy and composite epoxy materials. A more comprehensive discussion of these changes will be presented in the subsequent section.

**Figure 5:**
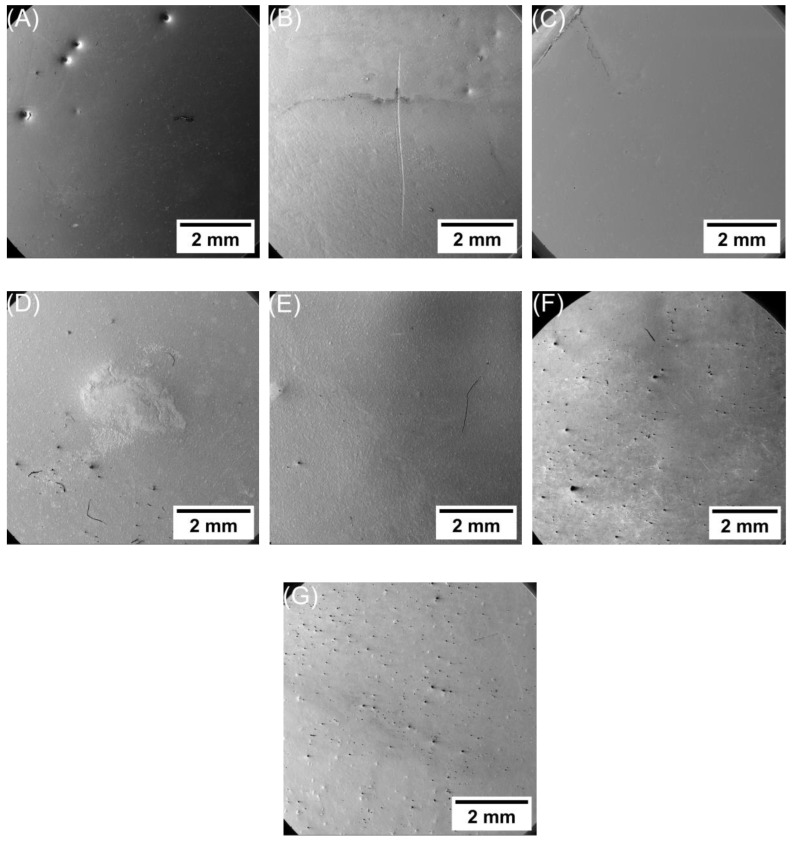
Full-view SEM micrographs for samples after applying 6 kV for 3 h: (**A**) unfilled epoxy; (**B**) epoxy/3 wt.% ${SiO}_{2}$ nanocomposites; (**C**) epoxy/5 wt.% ${SiO}_{2}$ nanocomposites; (**D**) epoxy/3 wt.% MgO nanocomposites; (**E**) epoxy/5 wt.% MgO nanocomposites; (**F**) epoxy/3 wt.% ${Al}_{2}O_{3}$ nanocomposites; (**G**) epoxy/5 wt.% ${Al}_{2}O_{3}$ nanocomposites.

**Figure 6:**
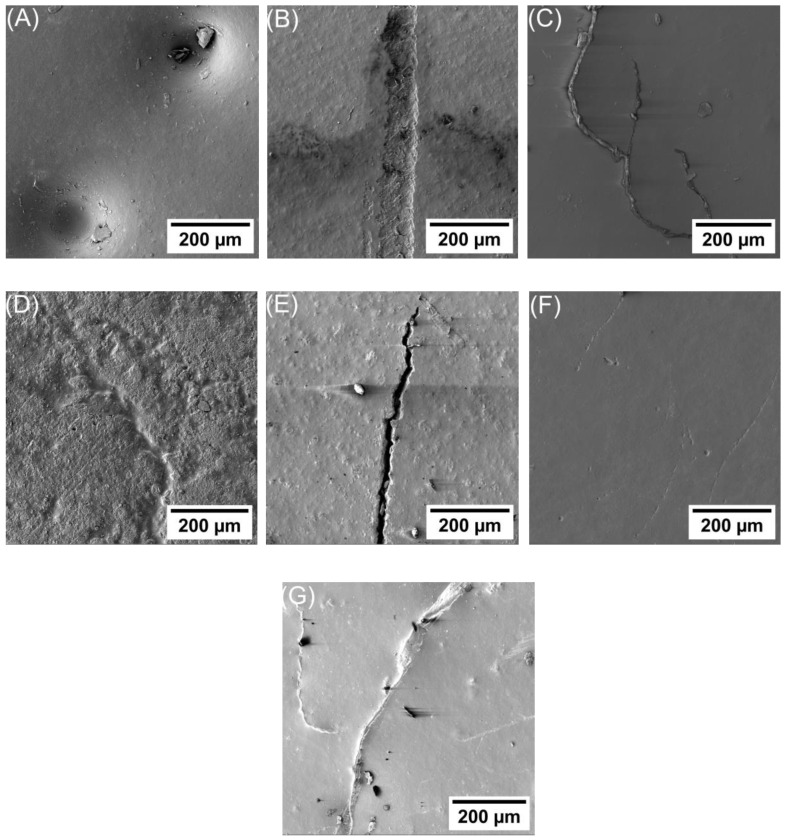
SEM micrograph for samples after applying 6 kV for 3 h with high magnification: (**A**) unfilled epoxy; (**B**) epoxy/3 wt.% ${SiO}_{2}$ nanocomposites; (**C**) epoxy/5 wt.% ${SiO}_{2}$ nanocomposites; (**D**) epoxy/3 wt.% MgO nanocomposites; (**E**) epoxy/5 wt.% MgO nanocomposites; (**F**) epoxy/3 wt.% ${Al}_{2}O_{3}$ nanocomposites; (**G**) epoxy/5 wt.% ${Al}_{2}O_{3}$ nanocomposites.

Figure 5 shows SEM micrographs that provide a comprehensive perspective of the samples, whereas Figure 6 presents a high-magnification SEM micrograph that shows a more detailed examination of the alterations in the samples after applying 6 kV for a duration of 3 h. The durability of the samples varies depending on the filler material used and its concentration. As shown in Figure 5A and Figure 6A, several semi-carbonized holes are formed in the unfilled epoxy, which indicates that the material completely breaks down at these locations after applying 6 kV for 3 h duration.

Figure 5B shows a sample of epoxy/3% ${SiO}_{2}$ nanocomposite with many holes along with erosion, indicating the early stages of crack formation and being more carbonized. Notably, the presence of nanoparticles has prevented further crack development. Additionally, observable in the Figure 5B is charring that traverses the erosion and extends along the sample. Figure 6B shows a more detailed view of the erosion and charring phenomenon. In Figure 5C, a deep crack (partial discharges) is apparent at the tip of the epoxy/5% ${SiO}_{2}$ nanocomposite sample, which is shown as a result of a weakening of this specific region by the applied voltage. Figure 6C shows the tree pattern of this crack, with nanoparticles effectively impeding its linear progression and its depth. However, in Figure 5D, a large area of erosion and tree formation is visible on the surface of the epoxy/3% MgO nanocomposite sample. The topography of this area is shown in Figure 6D. The epoxy/5% Mgo nanocomposite sample showed a low defect, except for the partial discharges that developed in the center of the sample, as shown in Figure 5E and Figure 6E. Moreover, in the sample of epoxy/3% Al_2_O_3_ nanocomposite, a large surface tree is observed, as shown in Figure 5F and Figure 6F. While the epoxy/5% Al_2_O_3_ nanocomposite sample showed excellent resistance to the applied voltage, its surface was free of any treeing, as shown in Figure 5G, but small trees appeared on the surface, as shown in Figure 6G.

**Figure 7:**
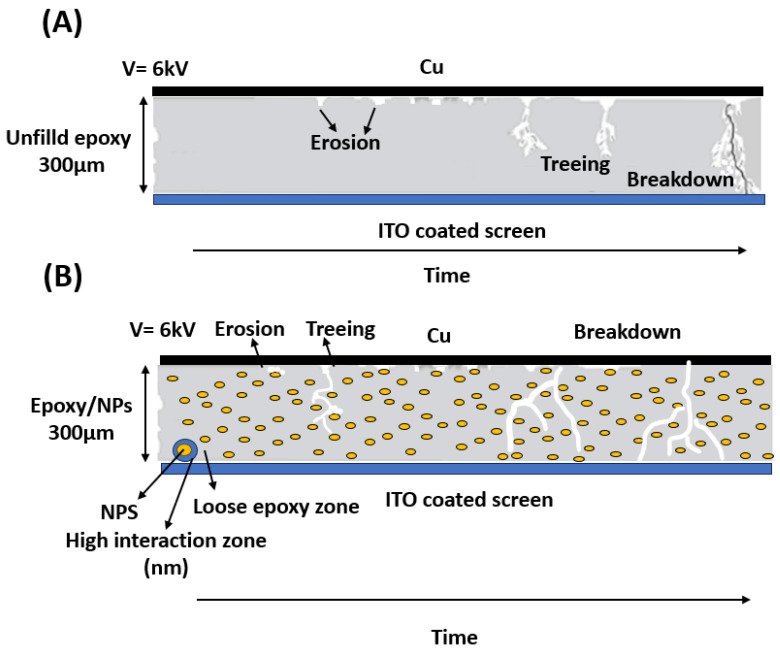
(**A**) Schematics diagram of the DC breakdown mechanism in (**A**) unfilled epoxy. (**B**) Epoxy nanocomposite.

The results obtained and shown in Figure 5 and Figure 6 can be used to interpret the results achieved and to explain the formation of holes and trees in unfilled and nanocomposite epoxy, as shown in the schematic diagram in Figure 7. Usually, the electrode–dielectric interface between cathodes and anodes has an almost uniform electric field interface [27,28]. Due to the quasi-homogeneous nature of the field, it is not feasible in this electrode configuration to pinpoint a distinct point of origin or an endpoint for the progression of damage. The damage may initiate anywhere between the two contacts [27,28]. However, the largest value of the field is at the edges, increasing the likelihood of degradation occurring at the edges. However, the application of high voltage over a long period of time can also contribute to a significant difference in the sample dielectric strength. This adheres to the idea of the global weakest link (GWL) reported by Flandin et al. [28]. This concept suggests that the damage can initiate anywhere between the two contact electrodes.

**Figure 8:**
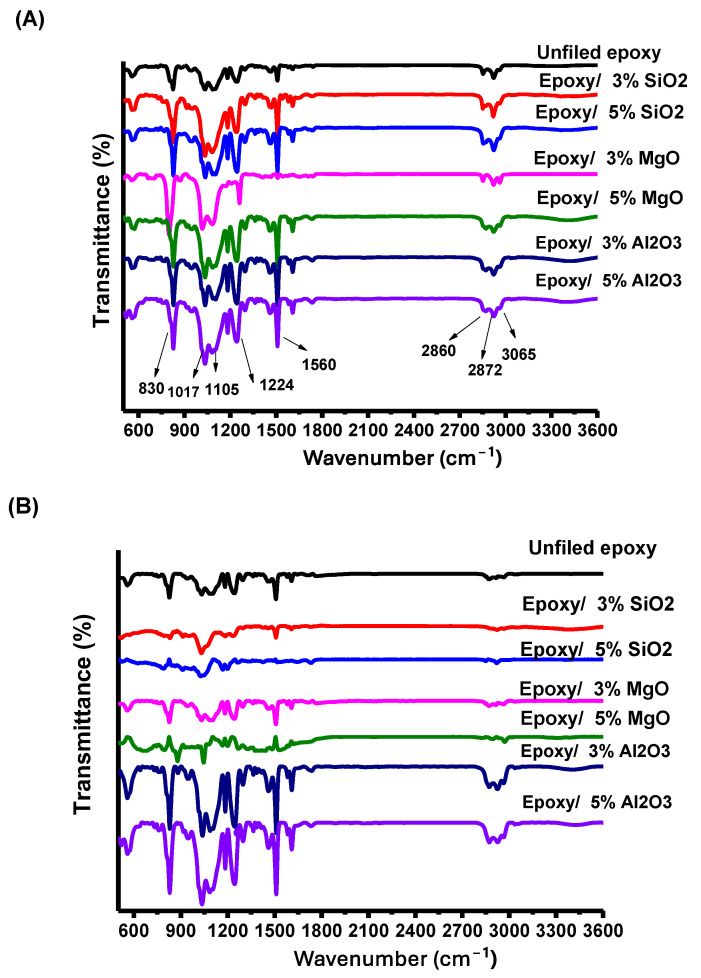
FTIR spectra of unfilled epoxy and epoxy/nanocomposite (**A**) before applying electric field (**B**) after applying electric field.

In an unfilled epoxy, erosion typically begins after a high voltage is applied starting in the areas where the electrical field exhibits the highest level of non-uniformity. Over time, the depth of erosion will increase which is referred to as a partial discharge. These discharges manifest themselves on the surface of the insulation material. In the next stage, the intensity of the partial discharge will continue to escalate, resulting in the widening of the erosion channel and the appearance of treeing. Finally, breakdown occurs as the corona discharge imposes electrical stresses above the threshold voltage, initiating the discharge and resulting in the gradual deterioration of the epoxy [4,7,9,12,16]. This deterioration occurs either as a result of the thermal energy released by the discharge or as a result of carbon bond disruption caused by the discharge’s impact [4,7,9,12,16]. The changes that occurred in the carbon bonds after applying a high voltage were studied using FTIR, as shown in Figure 8.

After that, erosion significantly accelerates with both increased voltage levels and time, and the discharges tend to aggregate, leading to the formation of deep pits inside epoxy resin. Each discharge releases more energy with increasing length, and erosion proceeds at a faster pace until these pits reach a critical length. At this point, narrow semi-carbonized channels tend to develop at the ends of these pits, often triggering complete breakdown, as shown in Figure 6A,B. These ultimate breakdown channels propagate when electrical stress surpasses inherent electric strength over a certain minimum distance [15]. Figure 7A shows a schematic diagram of the breakdown mechanism in unfilled epoxy.

Upon the introduction of NPs into the epoxy matrix, a discernible alteration in the epoxy’s morphology ensues and the encompassing layer around the nanoparticle consists of two regions: a high interaction zone, also called an interfacial nanolayer, and a loose polymer zone [29]. Figure 7B shows a schematic representation of an epoxy nanocomposite and illustrates the DC breakdown mechanism occurring within it. As the exposure to discharges continues, these channels become progressively deeper and wider, as shown in Figure 6D,E. However, their growth in any direction is impeded by the presence of filler particles. Consequently, these channels remain confined to the loose epoxy zone. The paths of erosion in filled samples take on a zigzag pattern like a tree as they navigate around the filler particles. At high voltages and over time, corrosion initiates within the loose epoxy zone located between the NPs, resulting in the formation of degradation channels. This is due to the increased resistance to degradation exhibited by both the high interaction zone and the NPs compared to the loose epoxy zone. Additionally, with the escalation of channel depth, nanoparticles function as barriers contributing to the cessation of degradation. If the nanoparticles (NPs) are well disturbed, they will create a larger interaction zone and reduce interparticle distances. This results in cutting these trees or increasing the tree paths as they extend away from the NP tree paths as they extend away from the NPs. This contributes to delaying the occurrence of DC breakdown, thus increasing the life of the sample. This result is supported by Figure 6B–G. Moreover, it is consistent with the literature [12,15,30].

In the case of epoxy/3 and 5 wt.% of ${SiO}_{2}$ nanocomposite, a reduction in the DC breakdown strength has been recorded as can be inferred from Figure 6B,C. In the cases of epoxy/3 wt.% and 5 wt.% of MgO nanocomposite, an increase in the strength of DC breakdown was shown in Figure 6D,E. However, it should be noted that this enhancement did not provide complete prevention from DC breakdown.

Considering that the samples were prepared using the same methodology, the improvement in breakdown strength of Epoxy/3 and 5 wt.% of ${Al}_{2}O_{3}$ nanocomposite can be attributed to the larger ${Al}_{2}O_{3}$ size compared to ${SiO}_{2}$ and MgO. Because of the high packing density, the accumulated charge was reduced and the threshold field at any point was reduced, and the space charge distribution was altered [9,27]. In addition, when the material is packed with NPs, the fillers act as a scattering site for an electric field and electrons can not gain momentum to be involved in the process of breakdown. Under high-voltage application, the ejected electrons are accelerated by the applied field colliding directly with the highly dispersed surrounding NPs and losing their momentum. It requires additional voltage to cause a catastrophic failure of the insulation and cause damage to the insulation [9,15]. Therefore, due to these aforesaid reasons, epoxy/${Al}_{2}O_{3}$ nanocomposite has improved breakdown strength compared to the unfilled epoxy, as shown in Figure 6F,G. A very similar effect was modeled by various researchers [9,15,27,30].

Figure 8A shows the main carbon bonds in the epoxy resin. Under the same conditions, the transmission spectra of the samples were obtained using a vacuum Fourier Transform Infrared (FTIR) Vertex 70v (Bruker, Billerica, MA, USA). The transmission spectrum of epoxy/nanocomposites, featuring distinct bands at 830 cm⁻¹ and 1224 cm⁻¹, was assigned to the bending of CH groups. Furthermore, the band observed at 3065 cm⁻¹ is attributed to the C-H stretching mode of the epoxide group. Additionally, the bands at 2957 cm⁻¹ and 2875 cm⁻¹ correspond to the -CH_2_ and -CH_3_ stretching vibration modes of aromatic and aliphatic chains, respectively [31] [NO_PRINTED_FORM]. The peak at 1105 cm⁻¹ can be attributed to CO stretching. Meanwhile, the band at 1017 cm⁻¹ is associated with COC in epoxy groups [31,32].

Figure 8B shows the changes that occurred in carbon bonds after exposure of the samples to high voltage. Upon comparing Figure 5A,B, it is evident that no significant changes were observed in the carbon bonds within the epoxy/3 and 5% ${Al}_{2}O_{3}$ nanocomposite. When observing the spectra, it is clear that there is a reduction or disappearance of peaks at 570, 1017–1105 cm⁻¹, and 2875–3065 cm⁻¹ in other nanocomposites with varying proportions.

### 4.2. Dielectric Properties

The dielectric measurements were conducted using a Quatro cryo-system, manufactured by Novocontrol Technologies GmbH & Co. KG, Montabaur, Germany, in conjunction with the Novocontrol Alpha-A analyzer. The conducted measurements encompassed a wide range of frequencies—from 10^−2^ Hz to 10^7^ Hz. The complex permittivity $(\hat{\varepsilon }$), a frequently employed parameter, can be expressed as follows [33]:

(1) $$\hat{\varepsilon }\;\left(\omega \right)=\varepsilon^{\prime }(\omega )+i\varepsilon^{\prime \prime }(\omega )$$

where $i=\sqrt{-1}$, ω is the angular frequency, while ε′ and ε″ denote the real and imaginary components of permittivity, respectively. Furthermore, the calculation of tan(δ) for a capacitor configured with two plates separated by an epoxy/nanocomposite is expressed as follows [22,33,34]:

(2) $$tan\delta =\frac{\varepsilon^{\prime \prime }\left(\omega \right)}{\varepsilon^{\prime }(\omega )}$$

**Figure 9:**
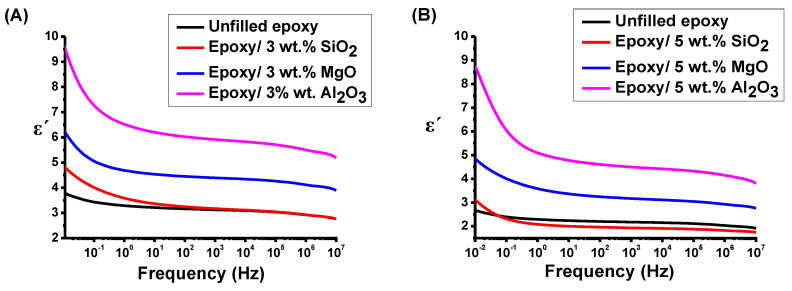

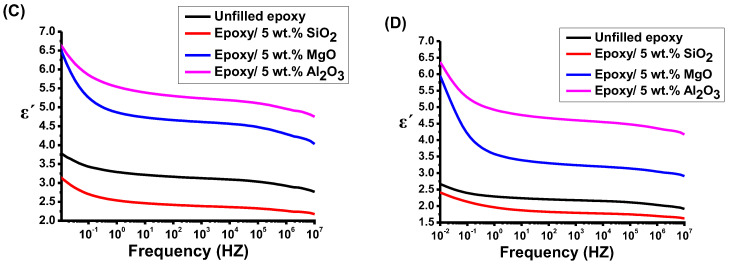
(**A**) ε′ for epoxy/3% NP nanocomposites. (**B**) ε′ for epoxy/3% NP nanocomposites after applying 6 kV. (**C**) ε′ for epoxy/5% NP nanocomposites. (**D**) ε′ for epoxy/5% NP nanocomposites after applying 6 kV.

Due to the limitations of the mobile charge generation and the mobility of charge carriers in polymeric insulators, arising from the incorporation of NPs in the epoxy, variations in electrical conductivity are anticipated. These variations are contingent upon changes in filler concentration and high-voltage exposure. Consequently, the electrical conductivity of NPs becomes a critical factor in influencing the changes in the tan(δ) value. The real part of the conductivity, denoted as σ′(ω), can be expressed as follows [22]:

(3) $$\sigma^{\prime }\left(\omega \right)=\omega \varepsilon_{0}\varepsilon^{\prime \prime }(\omega ),$$

where $\varepsilon_{0}$ is the permittivity of the free space. Figure 9 shows the permittivity variation as a function of frequency.

As shown in Figure 9A, at frequencies lower than 10^1^ Hz, the permittivity is influenced by the presence of orientable dipoles and their ability to align with the applied electric field [22,31]. At lower frequencies, the ε´ increases due to the effective orientation of all free dipolar functional groups within the epoxy chains. However, with an increase in the frequency of the electric field, the larger dipolar groups contribute less to the ε′. This is because they are unable to align at the same rate as the alternating field. Consequently, the dielectric constant of the epoxy system is decreased [34,35]. Before applying high voltage, the incorporation of NPs induced various alterations contingent upon the concentration. Specifically, adding 3 wt.% NPs to the epoxy resulted in the polarization bonds that are easily formed under the electrical field. This phenomenon leads to an increase in permittivity compared to the unfilled epoxy counterparts, as shown in Figure 9A. The epoxy/3 wt.% ${Al}_{2}O_{3}$ exhibited the highest ε′ value, primarily attributed to the significant accumulation of space charges [31,34,35].

Nevertheless, with 5 wt.% NP, a notable reduction in ε′ was observed compared with that of epoxy/3 wt.% NPs. This reduction can be attributed to the interfacial polarization, commonly known as the Maxwell–Wagner–Sillars (MWS) effect [36]. The MWS effect arises from the accumulation of charges at interfaces inside heterogeneous media characterized by diverse dielectric properties and conductivity, as shown in Figure 9C [34,35].

As depicted in Figure 9B,D, the concentration and size of the filler play a crucial role in the breakdown phenomenon [2,37]. A discernible sharp decrease in permittivity is evident for the unfilled epoxy and the epoxy/3 and 5 wt.% of ${SiO}_{2}$ nanocomposites, signifying the occurrence of collapse. In the case of the epoxy/3 wt.% MgO, the rate of permittivity decrease was 1.4, while it decreased to 0.7 in the case of the epoxy/5 wt.% of MgO nanocomposite. Conversely, the rate of change in permittivity for the epoxy/3 wt.% of ${Al}_{2}O_{3}$ was less than 0.5, while the rate of change in permittivity value was lower than that in the case of epoxy/5 wt.% ${Al}_{2}O_{3}$.

Upon the introduction of NPs into the epoxy matrix, a discernible alteration in the epoxy’s morphology ensues, driven by the interactions between the epoxy and the NPs. Several studies in the existing literature [12,29,35,37] reported that the interaction between the nanoparticle and the epoxy chains instigates the formation of an interfacial nanolayer encircling the nanoparticle [12,29,35,37]. Singha et al. [30] have proposed a dual-layer model, positing that the encompassing layer around the nanoparticle consists of two distinct regions: a tightly bound region and a loose polymer region. In this context, the tightly bound region, proximate to the nanoparticle, involves NPs forming hydrogen bonds with the polymer chain, thereby imposing restrictions on mobility within this specific region [12,29,37].

With 3 wt.% NPs, it was observed that the breakdown strength decreased as compared to unfilled epoxy. But, in this case, the number of particles is less and the interparticle distance is greater. Therefore, the volume fraction of the loose polymer layer is large. When high electric strength is applied, the transfer of charge carriers between the electrodes becomes easy through this loose polymer nanolayer, as shown in Figure 6B. Also, the filler particles have a permittivity higher than epoxy. So, there will be field enhancement at these particles leading to partial discharges. It has been reported earlier that the field enhancement by filler particles does not give a complete explanation of the reduced breakdown strength observed [29,37]. Despite the meticulous preparation of filled materials, the likelihood of voids being present in the vicinity of filler particles cannot be ruled out. These voids may contribute to further field enhancement [29,37]. The combined effect of surface discharges localized partial discharges at the particle sites, and the easy transfer of charge carriers through the loose epoxy layer can lead to the final breakdown of the epoxy [12,15,30].

A further increase in the filler loading to 5 wt.% NPs showed a further decrease in the breakdown strength of epoxy nanocomposite. In this case, the number of NPs is greater, and the distance between NPs is smaller. Therefore, the number of immobilized nanolayers will increase, leading to a reduction in the loose polymer regions. This along with a large number of NPs can obstruct the discharge path, as shown in Figure 6C,E,G.

**Figure 10:**
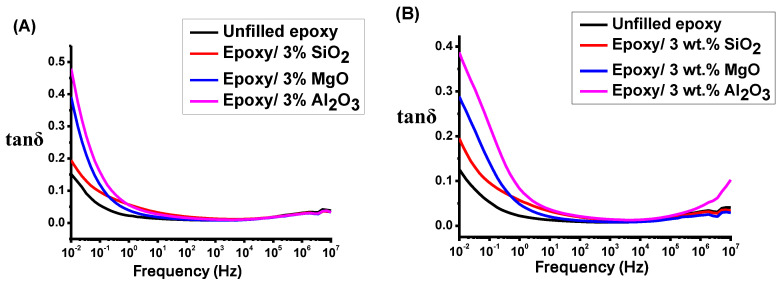

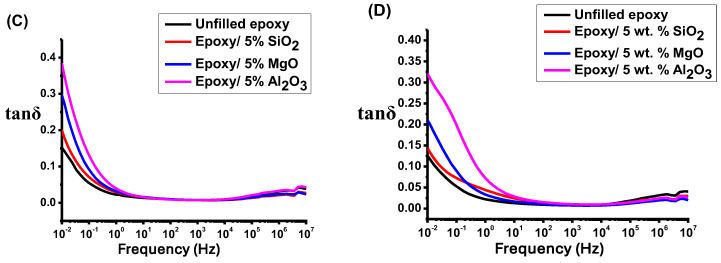
(**A**) tan(δ) for epoxy/3% NP nanocomposites. (**B**) tan(δ) for epoxy/3% NP nanocomposites after applying 6 kV. (**C**) tan(δ) for epoxy/5% NP nanocomposites. (**D**) tan(δ) for epoxy/5% NP nanocomposites after applying 6 kV.

Figure 10 shows the tan(δ) characteristics of unfilled and epoxy/nanocomposite samples before and after applying high voltage. As in Figure 10, tan(δ) exhibits elevated values at low frequencies compared to the high frequencies region, notably below $10^{1}$ Hz. Nevertheless, at a frequency higher than $10^{1}$ Hz, tan(δ) experiences a prompt reduction. The introduction of nanofillers at a concentration of 3 wt.% results in an increased heterogeneity in the epoxy composite, leading to the generation of free charges and a subsequent increase in tan(δ) compared to unfilled epoxy, as shown in Figure 10A. Conversely, at concentrations of 5 wt.% ${SiO}_{2}$ and MgO, a notable decrease is observed in tan(δ). This decline in tan(δ) values can be attributed to the creation of an interfacial nanolayer but at a lower percentage in epoxy/${Al}_{2}O_{3}$, as shown in Figure 10C.

In Figure 10B,D, the influence of high voltage on all samples is conspicuously apparent. It is observed that the tan(δ) value undergoes a significant reduction in both the unfilled epoxy and the epoxy/${SiO}_{2}$ nanocomposite, with a comparatively minor reduction in the epoxy/MgO nanocomposite. Conversely, the epoxy/${Al}_{2}O_{3}$ nanocomposite exhibits sustained stability under the impact of high voltage. The variation in breakdown severity observed in epoxy nanocomposites can be ascribed to multiple factors. One of them is the difference in permittivity between the ${Al}_{2}O_{3}$, MgO and ${SiO}_{2}$ NPs. The permittivity of ${SiO}_{2}$ NPs is lower as compared to that of MgO and ${Al}_{2}O_{3}$ NPs, as shown in Figure 9 [1,5,12,17]. So, the field enhancement in NPs will be lower than that in the case of epoxy/${SiO}_{2}$ silica nanocomposites. This leads to lower breakdown strength [16,38]. The low breakdown strength can be attributed to the elevated permittivity of ${Al}_{2}O_{3}$ [3,17].

The quantity of NPs, given the same filler loading, is greater for ${SiO}_{2}$ due to its smaller NP size compared to MgO NPs and ${Al}_{2}O_{3}$. The heightened presence of NPs can impede the penetration of charged carriers through the bulk of the epoxy silica nanocomposite sample, resulting in an increased value of the breakdown strength at a higher filler loading of 5 wt.% [3,4,37,39]. However, the presence of NPs is reported to enhance the ionization process within the sample, influencing space charge accumulations [40]. The type of filler plays a significant influence on these accumulations. Additionally, it has been documented that a higher content of nano-sized ${SiO}_{2}$ NPs (5 wt.%) in epoxy can lead to more intricate charge accumulation [40]. This complexity may contribute to the observed reduction in the breakdown strength of epoxy silica nanocomposites at higher filler loadings, as shown in Figure 6B,C.

As another result, Figure 10A–D show that the relaxation peak occurred at the same frequency for all samples (in the range of $5\times 10^{-6}$ Hz). This means that the relaxation time is constant for all samples at room temperature. Moreover, relaxation at high frequency is associated with β-relaxation. It also shows that the type and size of the filler, and the electric field have not strongly influenced the relaxation time at room temperature, while temperature is a major factor in changing the relaxation time based on the effect of the filler material and electron transport dependent on temperature. This will be discussed in detail in future work.

**Figure 11:**
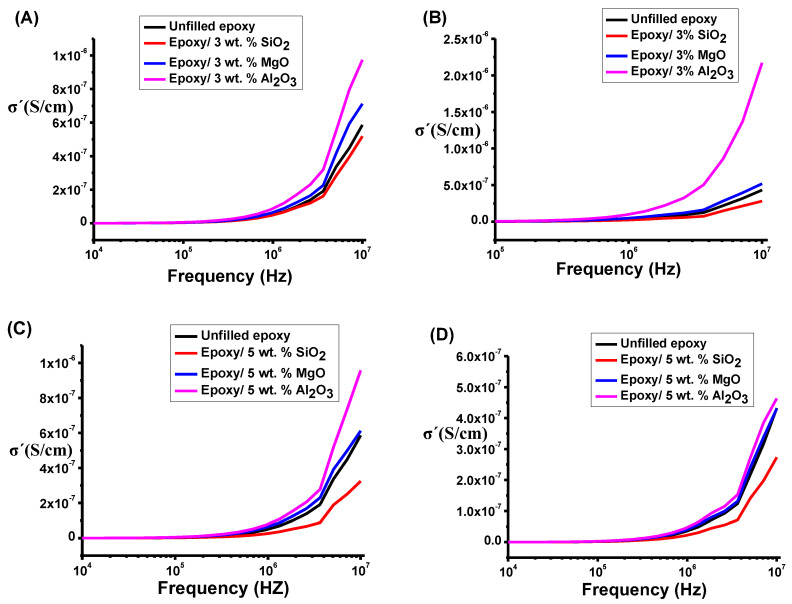
(**A**) σ′ for epoxy/3% NP nanocomposites. (**B**) σ′ for epoxy/3% NP nanocomposites after applying 6 kV. (**C**) σ′ for epoxy/5% NP nanocomposites. (**D**) σ′ for epoxy/5% NP nanocomposites after applying 6 kV.

In a broader context, the conductivity observations represented in Figure 11 are in accordance with the results expounded in Section 4.1 concerning the tan(δ). Figure 11 shows the conductivity of the epoxy composite before the application of 6 kV. Notably, the epoxy composite containing 3 wt.% NPs demonstrates the highest levels of electrical conductivity, as shown in Figure 11A. This can be attributed to the increase in space charge within the sample. In Figure 11B, a reduction in conductivity is evident for both the unfilled epoxy and epoxy/5 wt.% ${SiO}_{2}$, and epoxy/MgO, attributable to the occurrence of DC breakdown strength. Conversely, the epoxy/5 wt.% ${Al}_{2}O_{3}$ composite exhibited stability in the conductivity value.

This observation is consistent with findings reported in the literature [1,4,22,38]. In Figure 11B, the conductivity value of the epoxy/${SiO}_{2}$ and MgO decreased compared to Figure 11A. This reduction is attributed to the formation of immobilized nanolayers. The absence of an interfacial nanolayer in the epoxy/5 wt.% ${Al}_{2}O_{3}$ is attributed to the lower number of ${Al}_{2}O_{3}$ NPs compared to ${SiO}_{2}$ and MgO NPs. Consequently, the ${Al}_{2}O_{3}$ NPs remain dispersed and distant from each other [1,4,22,38]. It is noteworthy that the nonlinear conductivity is attributed to the presence of grain boundaries and the combined influence of both DC and AC conductivity [41]. Numerous sources in the literature have investigated this phenomenon, offering detailed discussions on the subject [1,4,22,38].

Despite the presence of an interfacial nanolayer in epoxy/5 wt.% ${SiO}_{2}$ and MgO nanocomposites, the breakdown occurred to a lesser extent in the epoxy/MgO nanocomposite, as shown in Figure 11D [39,42,43]. This discrepancy can be attributed to the distinct thermal conductivity of each material [41]. The potential enhancement in DC breakdown strength may arise from the increased conductivity of the epoxy/5 wt.% ${SiO}_{2}$ and MgO nanocomposite. This elevated conductivity facilitates more efficient dissipation of generated heat, resulting in a higher breakdown strength. The interphase region near the nanoparticle, as reported by Zhang et al. [42], is noted to be conductive. As the conductive interphase regions overlap, the neat epoxy region diminishes, providing a more accessible conducting path for charge transfer and consequently reducing the breakdown strength [44]. No breakdown was observed in the epoxy/${Al}_{2}O_{3}$ nanocomposite, as shown in Figure 4F,G.

## 5. Conclusions

The present study aims to use FEM analysis to study the DC breakdown strength of ${Al}_{2}O_{3}$, MgO, and ${SiO}_{2}$ epoxy nanocomposites and to assess changes in their electrical properties. The experiments included applying adjustable voltages of 6 kV to the samples for 3 h and monitoring the emission currents under high vacuum conditions up to $10^{-6}$ mbar. The results showed that the emission current varied depending on the nanocomposite material. Unfilled epoxy and those containing 3 and 5 wt.% ${SiO}_{2}$ nanocomposite showed a reduction in current, while composites with 3 and 5 wt.% of MgO nanocomposite initially had higher currents but decreased over time. However, epoxy with 3% and 5 wt.% of ${Al}_{2}O_{3}$ nanocomposite maintained a stable current.

The DC breakdown strength investigation revealed a reduction in epoxy/3 wt.% and 5 wt.% ${SiO}_{2}$ nanocomposites, and an increase in epoxy/3 wt.% and 5 wt.% MgO nanocomposites, although without complete prevention. The uniform patterns in the filled samples were observed at two stages, including erosion, degradation channel formation, and particle displacement. Epoxy/3 and 5 wt.% of ${Al}_{2}O_{3}$ nanocomposites exhibited high breakdown strength due to dispersed-sized alumina particles, reducing charge accumulation and enhancing breakdown resistance.

The present study emphasized the critical role of filler concentration, size, permittivity, and thermal conductivity in the breakdown phenomenon. The introduction of nanoparticles induced morphological changes and formed interfacial nanolayers. The breakdown strength decreased with 3 wt.% filler loading due to voids, while with 5 wt.% loading, it increased immobilized nanolayers, which hindered discharge paths.

## Figures and Tables

**Table 1:** The emission current (nA) values for all samples.

Time (min) Sample	1	90	180
Unfilled epoxy	0.30	0.15	0.002
Epoxy/3 wt.% ${SiO}_{2}$	0.14	0.09	0.045
Epoxy/5 wt.% ${SiO}_{2}$	0.21	0.12	0.085
Epoxy/3 wt.% MgO	0.45	0.24	0.100
Epoxy/5 wt.% MgO	0.48	0.34	0.081
Epoxy/3 wt.% ${Al}_{2}O_{3}$	0.23	0.21	0.200
Epoxy/5 wt.% ${Al}_{2}O_{3}$	0.25	0.23	0.220

**Table 2:** Breakdown strength and the time it took for the applied electric field to cause the collapse.

Sample	Breakdown Strength (V/m)	Time (min)
Unfilled epoxy	(2 × 10^6^)	90
Epoxy/3 wt.% ${SiO}_{2}$	(2 × 10^6^)	90
Epoxy/5 wt.% ${SiO}_{2}$	(2 × 10^6^)	180
Epoxy/3 wt.% MgO	(2 × 10^6^)	180
Epoxy/5 wt.% MgO	(2 × 10^6^)	180
Epoxy/3 wt.% ${Al}_{2}O_{3}$	-	-
Epoxy/5 wt.% ${Al}_{2}O_{3}$	-	-

## Data Availability

Data are contained within the article.
